# Lateral epicondyle osteotomy results in improved radiologic and functional outcomes in severe lateral tibial plateau fractures: a retrospective cohort study

**DOI:** 10.1186/s13018-025-05775-3

**Published:** 2025-04-10

**Authors:** Hendrik Fahlbusch, P. Behrendt, A. Becker, C. Arras, H. Gablac, J. Frings, M. Hoffmann, M. Krause, K. H. Frosch

**Affiliations:** 1 Department of Trauma and Orthopaedic Surgery, University Medical Center Hamburg-Eppendorf, Hamburg, Germany; 2 Department of Orthopedics and Traumatology, University Medical Center Schleswig-Holstein, Campus Kiel, Kiel, Germany; 3 Department of Anatomy, Christian-Albrechts-University, Kiel, Germany; 4 Department of Trauma Surgery, Orthopedics and Sportsorthopedics, Asklepios St. Georg, Hamburg, Germany; 5 Department of Trauma Surgery, Orthopaedics and Sports Traumatology, BG Hospital Hamburg, Hamburg, Germany

**Keywords:** Tibial head fracture, Extended lateral approach, Epicondyle, Osteotomy, 10-Segment classification

## Abstract

**Background:**

This study evaluated the clinical and radiological outcomes of lateral tibial plateau fractures involving the central and postero-lateral regions, comparing an extended lateral approach with lateral epicondyle osteotomy (ECO) to a conventional approach without an extention (No-ECO).

**Methods:**

A retrospective cohort study was conducted at two centers, examining complex lateral tibial plateau fractures treated with either an extended lateral approach with ECO or without it. Only AO/OTA type B3/C3 fractures involving the antero-latero-central (ALC) and postero-latero-central (PLC) segments were included. Fracture reduction quality was assessed via post-operative CT scans, and clinical outcomes and complications were evaluated over a minimum of 24-month follow-up.

**Results:**

A total of 110 patients (mean age: 51.3 ± 11.1 years) were included, with an average follow-up of 52.7 ± 16.9 months. The ECO group (*n* = 56) consisted of more severe injuries, indicated by higher external fixator use (48.2% vs. 22.2%, *p* = 0.0044) and additional affected segments. Postoperative CT scans revealed that the ECO group had significantly less fracture step-off (0.8 mm vs. 3.0 mm, *p* = 0.0002) and angulation at the ALC/PLC (8.1° vs. 20.1°, *p* = 0.0002) segment and PLC/PLL (postero-latero-lateral) (2.2° vs. 7.5°, *p* = 0.02) segments. Clinically, the ECO group achieved superior IKDC scores (71.7 vs. 63.7, *p* = 0.0097). A negative correlation was found between postoperative ALC/PLC depression and IKDC scores (*r*=-0.36, *p* = 0.0002).

**Conclusion:**

Patients treated with ECO had a significantly better clinical and radiologic postoperative outcomes, with the quality of fracture reduction positively correlating with the clinical IKDC score. This was achieved despite more severe injuries, as indicated by higher external fixator use and number of affected segments.

**Level of evidence:**

III Retrospective Cohort Study.

**Trial registration:**

The study was retrospectively registered and conducted according to the guidelines of the Declaration of Helsinki and approved by the local Ethics Committee (PV7319).

**Supplementary Information:**

The online version contains supplementary material available at 10.1186/s13018-025-05775-3.

## Introduction

Tibial plateau fractures, particularly type 41-B3 and 41-C3 as classified by AO/OTA, commonly involve the posterior-latero-central (PLC) segment, postero-latero-lateral (PLL) segment and the posterior aspect of the antero-latero-central (ALC) segment, according to the 10-segment classification system by Krause et al. [18, 31].

Adequate reduction of these segments is crucial for preventing post-traumatic deformities and osteoarthritis, which can significantly impair functional outcomes and quality of life. Maintaining a postoperative intraarticular joint irregularity of less than 2.5 mm is associated with improved range of motion, reduced pain, and higher KOOS scores [29–31]. Despite advancements in open reduction and internal fixation techniques, postoperative CT-Scans reveal malreductions in up to 32.3% of cases, particularly in the posterior quadrants of the lateral plateau [26].

Factors such as age, body mass index, fracture type, use of bone graft or bone graft substitute and the use of locking plates do not predict malreduction [26]. An intraoperative 3D scan serves as a retrospective examination, useful for additional control and documentation of anatomic reduction after fracture reduction and (temporary) fixation in complex fractures. However, its value as a reduction tool is limited and it cannot replace direct visualization [17, 26].

If the central area of the lateral plateau is involved, a complete visualization of the fractured articular surface is difficult and cannot be reached by standard approaches in most cases. The classic anterolateral approach provides access to only 36.6% of the anterior and lateral articular surface, resulting in a 16.6% malreduction rate [19, 26]. Recent studies have investigated various surgical approaches to improve direct visualization and improved reduction of the PLC segment [2, 11, 14, 20].

First, rim plating of posterolateral fracture fragments through a modified anterolateral approach in tibial plateau fractures necessitates releasing the lateral collateral ligament (LCL) [7]. This procedure causes irreversible LCL damage due to necessary retraction for exposure and is not recommended. Second, lateral rim osteotomy of the lateral tibial plateau can expose and reduce central segments [35], but is an additional intraarticular osteotomy. However, it often provides insufficient visualization of the articular surface post-reduction, risking malreduction. Third, fibular head osteotomy allows visualization of about 87% of the articular surface [20, 23, 32]. This technique risks extensive soft tissue damage, peroneal nerve injury, disruption of the proximal tibiofibular syndesmosis, and unstable osteosynthesis [12].

A more straightforward and soft tissue-preserving procedure is lateral epicondyle osteotomy (ECO). This method avoids peroneal nerve exposure and allows stable refixation with two lag screws [37]. Including both the LCL and popliteus tendon footprint in the osteotomy significantly improves visualization of the lateral plateau, covering 83% of it [19, 20, 23, 32, 35]. Cadaveric studies show this method enhances radiological reduction quality, though clinical data is limited [17, 18, 20, 26].

This study aims to compare the outcomes of lateral tibial plateau fractures involving the ALC and PLC segments treated with either the extended lateral approach using additional ECO or standard approaches without extension. We hypothesize that in tibial plateau fractures involving the ALC and PLC segments, a surgical approach incorporating epicondyle osteotomy can achieve superior fracture reduction and ultimately leading to better clinical outcomes than an extended lateral approach alone. Additionally, we hypothesize that the morbidity associated with ECO does not negatively impact clinical outcomes.

### Study patients

**Fig. 1:**
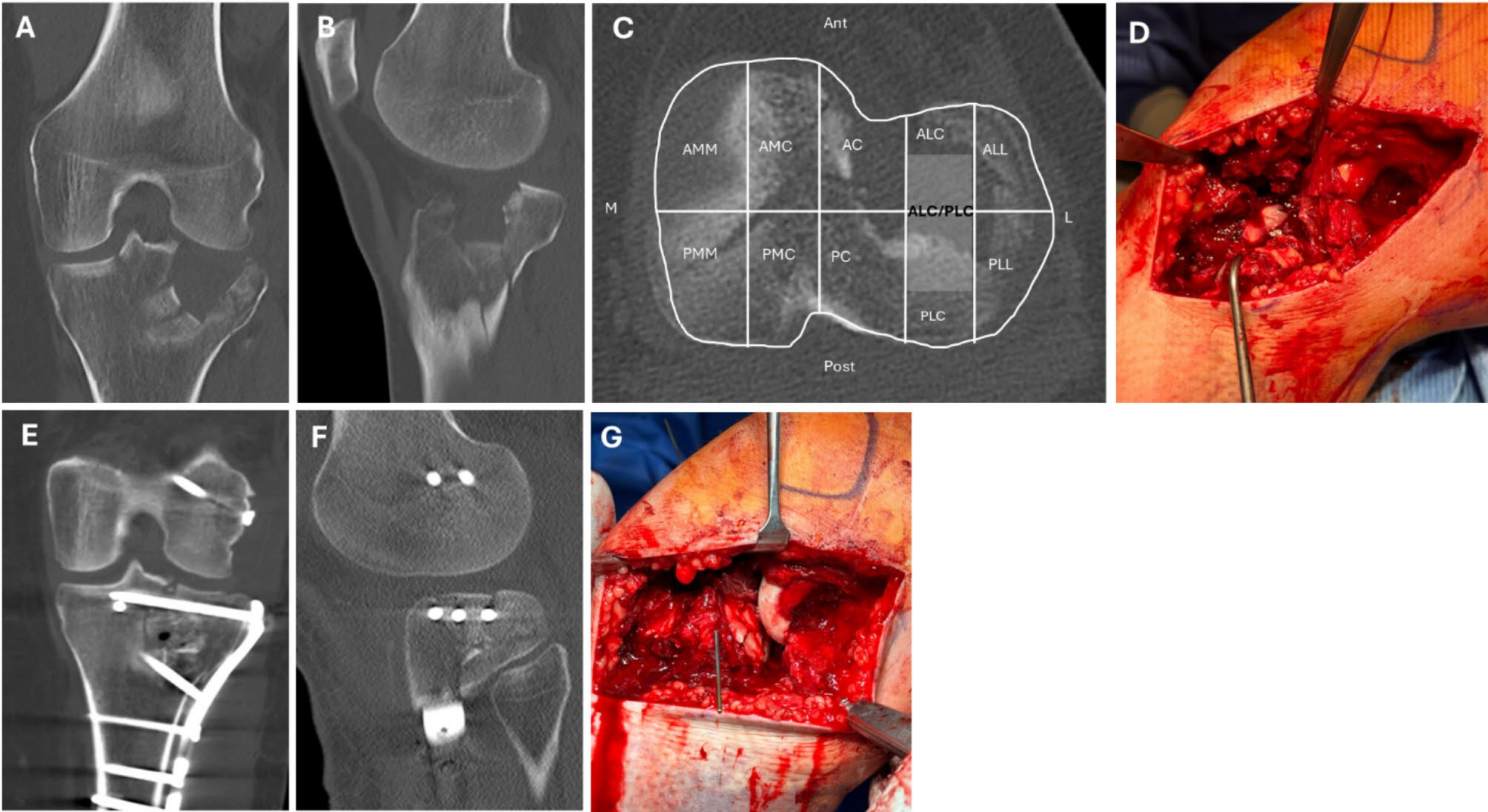
Exemplary cases of a patient treated with lateral epicondyle Osteotomy. A 47-year-old female with an AO/OTA type C3 fracture, involving all segments except the anteromedio-medial **(**AMM**)** segment, as defined by the 10-segment classification. The hatched area indicates the crossing of the anterior-lateral column **(**ALC**)** and posterior-lateral column (PLC). Preoperative CT scans demonstrate the fracture pattern in coronal (**A**), sagittal **(B**), and axial **(C**) views. Postoperative CT scans show successful reduction and allogenous bone grafting of the articular surface in coronal (**E)** and sagittal (**F**) views. Intraoperative images include the fracture prior to reduction after epicondyle osteotomy (**D)** and temporary fixation with K-wires (**G**)

The in-house trauma register of tibial plateau fractures at two level-I trauma centers was retrospectively reviewed between 2018 and 2021. All tibial plateau fractures treated via an extended lateral approach were identified and classified using the OTA classification and 10-segment classification systems (see Fig. 1B) [18, 25]. Inclusion criteria encompassed fracture patterns involving the at least the ALC and PLC segment. Additionally, the fractures were required to be classified as either OTA Type B3 or C3. Exclusion criteria included patients under 18 years of age, individuals with open fractures, patients with polytrauma, those undergoing primary endoprosthesis treatment, and cases lacking both pre- and postoperative CT scans. Furthermore, complex bicondylar fractures necessitating an extended medial approach or medial epicondyle osteotomy were excluded, although fractures exhibiting a single medial split type were included.

### Surgical management and postoperative treatment

All fractures were stabilized using internal plating through standard anterolateral or modified posterolateral approaches, as previously described [13, 34]. Medial condylar fractures were addressed via a posteromedial approach with buttress plating. Postoperative lower leg CT scans were performed within the first 72 h to assess joint congruency (see Fig. 1D-F). Physical exercise commenced for alle patients 48 h post-surgery, starting with continuous passive joint motion in a supine position. Peripheral nerve block anaesthesia was administered as needed. All patients were advised to limit weight-bearing to 20 kg and restrict their range of motion (extension/flexion: 0°/0°/90°) for six weeks.

### Surgical technique

Access to the ALC and PLC segment was achieved using an extended anterolateral standard approach or a modified posterolateral approach including a two window technique, as described by Frosch et al. [13], or a combination of both.

Intraarticular access was obtained by dissecting the meniscotibial ligament in the anterolateral quadrant of the tibial plateau, followed by applying varus stress to visualize the articular surface (submeniscal approach). The decision regarding the necessity of an additional ECO to enhance visualization was determined at random by the surgeon, encompassing the femoral insertion of the lateral collateral ligament and the popliteus tendon [21]. All patients provided consent for the potential use of ECO. When an additional posteromedial approach was necessary, it was performed concurrently with the posterolateral approach. For cases utilizing the anterolateral approach, the surgeon decided whether to prioritize the posteromedial approach or perform it subsequently. Temporary fixation was achieved using K-wires, reduction clamps, and lag screws when feasible. Central meniscal subluxation was performed if necessary to improve visualization of the lateral plateau [15]. Final fixation involved the use of lag screws, subcortical screws employing the jail technique, and an anterolateral buttress plate. After reduction and fixation, the epicondyle osteotomy was reduced and secured with two cancellous screws.

### Radiological analysis

Reduction quality was postoperatively assessed by computer tomography using multiplanar reconstructed scans in all patients. Fracture steps, comminution, and fracture gaps were analyzed at the ALC/PLC and PLC/PLL segments. Clinically relevant comminution, defined by Rosteius et al., was identified as areas measuring at least 381.00 mm² [30]. The medial-to-lateral width of the tibial plateau was measured at its widest diameter post-reduction. The Radiological Rasmussen Score was applied to evaluate postoperative reduction quality.

### Clinical follow-up evaluation and complication analysis

Follow-up examinations were conducted at a minimum of two years post-surgery. Subjective and functional outcomes were assessed using the clinical Rasmussen score, the International Knee Documentation Committee (IKDC) subjective knee form score and Lysholm-Score. Early complications (within 3 weeks postoperatively) were defined as a fracture step > 2 mm, gap size > 5 mm, nerve damage, and delayed wound healing. Late complications (> 3 weeks postoperatively) were defined as subjective lateral varus instability, arthrofibrosis and conversion to total knee arthroplasty (TKA).

### Statistical analysis

Descriptive statistics were utilized to summarize the data. The study population was divided into two groups: patients who underwent ECO and patients who did not undergo epicondyle osteotomy (No-ECO). Normal distribution was assessed using the Shapiro–Wilk test. Statistical significance was determined using a two-tailed T-test or the Kolmogorov-Smirnov`s Test, as appropriate. The Chi-square test was used to evaluate the significance of associations between categorical variables by comparing observed and expected frequencies.

In addition, a subgroup analysis was performed comparing surgical approaches, fracture patterns, and OTA/AO classification. ANOVA was used to compare the means of multiple groups (surgical approaches) to determine if there were statistically significant differences between them.

Analysis was performed using GraphPad Prism 8 (San Diego, CA, USA), with a significance level of α = 0.05 or less considered statistically significant.

## Results

**Fig. 2:**
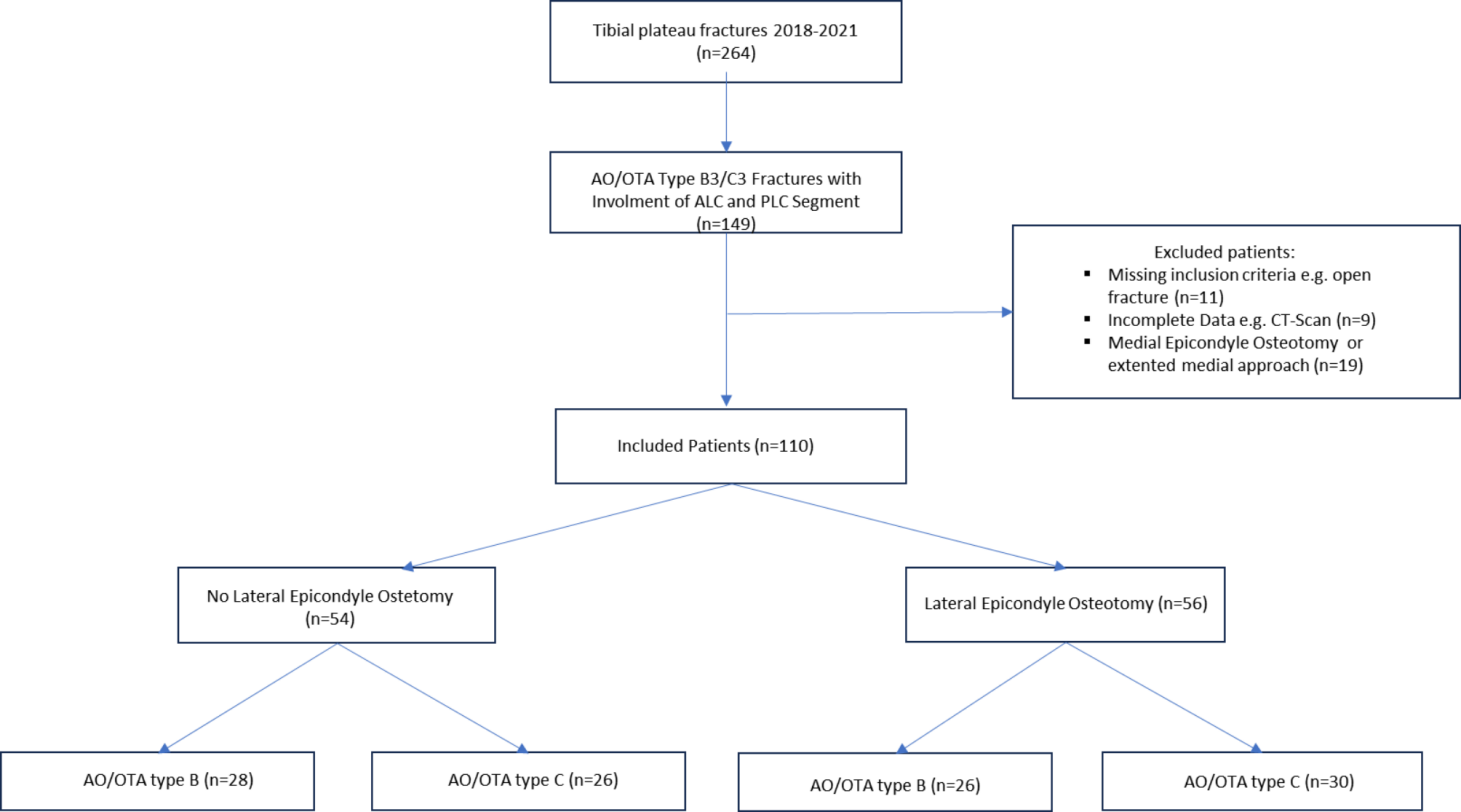
Flowchart patient selection. Flowchart showing the process of patient selection. Ultimately 110 patients were included

A total of 110 patients were ultimately included out of which 56 (50.1%) were treated by lateral ECO and 54 (49.9%) without ECO (see Fig. 2, Flowchart). All included patients were evaluated radiologically while 82 patients (74.5%) were available for clinical follow-up at a mean of 52.7 ± 16.9 months (Range 24–84 months). An analysis of baseline characteristics (demographic and preoperative data) of patients lost to follow-up showed no significant differences between the ECO and NoECO groups.

### Patient demographics

The demographic characteristics of the cases are presented in Table 1. There were significantly more external fixators prior to ORIF in the ECO group, but otherwise no differences in demographic characteristics between the two groups.

**Table 1:** Demographics

Parameters	ECO (*n* = 56)	No-ECO (*n* = 54)	Total (*n* = 110)	*p*-value
Age (in years) *	51.3 ± 11.1	56.2 ± 12.5	53.7 ± 8.8	n.s.
Female §	25 (52.1)	23 (47.9)	48 (49.2)	n.s.
Clinical Follow-Up (months) §	52.7 ± 16.9	55.1 ± 14.4	54.1 ± 15.4	n.s.
Comorbidities §				n.s.
• Smokers	6 (10.7)	9 (16.7)	15 (13.6)	
• Arterial Hypertension	7 (12.5)	10 (18.5)	17 (15.5)	
• Diabetes Mellitus	2 (3.6)	1 (1.9)	3 (2.7)	
• Asthma	4 (3.6)	2 (3.7)	6 (5.4)	
BMI [kg/m²] *	28.1 ± 7.1	27.1 ± 4.9	27.6 ± 6.1	n.s.
External Fixator before ORIF §	27 (48.2)	12 (22.2)	31 (35.5)	**0.0044**
Hospital-stay (in days) *				n.s.
• Total time	19.7 *±* 13.7	17.1 ± 10.1	18.4 *±* 12.1	
• Following surgery	12.1 *±* 11.6	10.1 ± 11.6	11.1 *±* 9.5	
Concomitant Injury Knee §	10 (15.4)	15 (23.1)	25 (38.5)	n.s.
• Cruciate Ligament	11 (19.6)	8 (14.8)	11 (17.3)	
• Collateral Ligament	7 (12.5)	5 (9.3)	15 (13.6)	
• Lateral Meniscus	24 (42.9)	26 (48.1)	50 (36.9)	

*Mean ± SD; § n (%); BMI body mass index, ORIF open reduction and internal fixation, n.s. non-significant; To compare ECO and No-ECO Student`s t-Test or Chi-Square Test were performed

### Preoperative characteristics

Preoperative characteristics showed an almost equal distribution of AO/OTA type B and C fractures. The most common segments involved were ALC/PLC (100%), PLC/PLL (86.4%) and AC/PC (60.9%) with significantly more cases in patients who underwent ECO (Table 2).

**Table 2:** Preoperative characteristics

Parameters	ECO (*n* = 56)	No-ECO (*n* = 54)	Total (*n* = 110)	*p*-value
Type of injury §				n.s.
• OTA Type B3	26 (46.4)	28 (51.9)	54 (49.1)	
• OTA Type C3	30 (53.6)	26 (48.1)	56 (50.9)	
Number of involved Segments*	5.8 ± 1.9	5.2 ± 1.9	5.5 ± 1.9	n.s.
Affected Segments §				
• ALC/PLC	56 (100)	54 (100)	108 (100)	n.s.
• PLC/PLL	53 (94.6)	42 (77.8)	95 (86.4)	**0.01**
• AC/PC	40 (71.4)	27 (50)	67 (60.9)	**0.0213**
• AMC/PMC	11 (19.6)	8 (14.8)	19 (17.3)	n.s.
• PMC/PMM	3 (5.4)	4 (7.4)	7 (6.4)	n.s.
• Medial Split	11 (19.6)	8 (14.8)	19 (17.3)	n.s.

§ n (%); *Mean ± SD, ALC antero-latero-central, AMC antero-medio-central, PLC postero-latero-central, PLL postero-latero-lateral, PMC postero-medio-central, AC antero-central, PC postero-central, n.s. non-significant; Bold text indicating *p* < 0.05. To compare ECO and No-ECO Chi-Square Test was performed

### Operative characteristics

Various intraoperative positionings and approaches were used, predominantly supine (71.8%), followed by prone (15.5%) and lateral (10.9%). There was no difference in positioning, approaches, and number of plates between groups. Detailed information regarding the surgical approach, patient positioning, and technique used to expose the lateral plateau is provided in Table 3.

**Table 3:** Operative characteristics

Parameters	ECO (*n* = 56)	No-ECO (*n* = 54)	Total (*n* = 110)	*p*-value
Time (in min) *	295.9 ± 125.8	268.5 ± 106.8	282.5 ± 117.1	n.s.
Positioning §				n.s.
• Supine	36 (64.3)	43 (79.6)	79 (71.8)	
• Prone	12 (21.4)	5 (9.3)	17 (15.5)	
• Prone and Supine	4 (7.1)	2 (3.7)	6 (5.5)	
• Side	8 (14.3)	4 (7.4)	12 (10.9)	
Lateral Approach §				n.s.
• Anterolateral	34 (60.1)	42 (77.8)	76 (69.1)	
• Posterolateral	17 (30.4)	11 (20.4)	28 (25.5)	
• Combined	5 (8.9)	1 (1.9)	6 (5.5)	
Additional medial approach for ORIF §				n.s.
• Anteromedial	10 (17.9)	13 (24.1)	17 (20.9)	
• Posteromedial	14 (25.0)	10 (18.5)	30 (30.9)	
Number of Plates §				n.s.
• Single	40 (71.4)	42 (77.8)	82 (74.5)	
• Double	16 (28.6)	16 (29.6)	32 (29.1)	
Jail Screw §	34 (60.1)	24 (44.4)	60 (54.5)	n.s.

*Mean ± SD; § n (%); ORIF open reduction and internal fixation, n.s. non-significant; Bold text indicating *p* < 0.05. To compare ECO and No-ECO Student`s t-Test or Chi-Square Test were performed

### Radiological results

The radiological findings (Table 4) revealed that postoperative fragment depression (*p* = 0.0002) and angulation (*p* < 0.0001) at the ALC/PLC segment crossing were significantly greater in fractures treated without ECO compared to those treated with ECO. No significant differences were observed between the groups concerning preoperative parameters and other postoperative parameters.

**Table 4:** Radiological results

Parameters *	ECO (*n* = 56)	No-ECO (*n* = 54)	Total (*n* = 110)	*p*-value
Preoperative maximum fragment depression (in mm)	12.0 ± 7.7	9.9 ± 8.3	11.0 ± 8.0	n.s.
Preoperative maximum fragment angulation (in °)	31.3 ± 19.8	28.1 ± 24.9	29.8 ± 22.4	n.s.
Postoperative depression (in mm)				
• ALC/PLC segment crossing	0.8 ± 1.1	3.0 ± 3.2	1.8 ± 2.5	**0.0002**
• PLC and PLL segment	0.4 ± 1.2	1.6 ± 2.7	1.0 ± 2.1	n.s.
Angulation (in °)				
• ALC/PLC segment crossing	8.1 ± 13.5	20.1 ± 18.4	13.8 ± 18.0	**0.0002**
• PLC and PLL segment	2.2 ± 11.8	7.5 ± 20.1	6.7 ± 16.0	**0.0378**
Gap (in mm)	2.1 ± 4.3	3.5 ± 7.0	2.7 ± 5.7	n.s.
Tibial slope (in °)	8.8 ± 4.1	7.4 ± 5.1	8.2 ± 4.7	n.s.
Width of femur and tibia (in mm)				
• Femur	82.1 ± 7.0	81.9 ± 12.1	82.1 ± 9.8	n.s.
• Tibia	79.7 ± 7.3	77.1 ± 10.7	78.7 ± 9.1	n.s.
• Ratio (tibia/femur)	0.9 ± 0.05	0.9 ± 0.0	0.96 ± 0.0	n.s.
Radiological Rasmussen-Score	14.8 ± 3.3	13.8 ± 2.9	14.3 ± 3.2	n.s.

*Mean ± SD; n.s. non-significant; Bold text indicating *p* < 0.05. To compare ECO and No-ECO Student`s t-Test or Kolmogorov-Smirnov`s Test was performed

### Clinical results

**Fig. 3:**
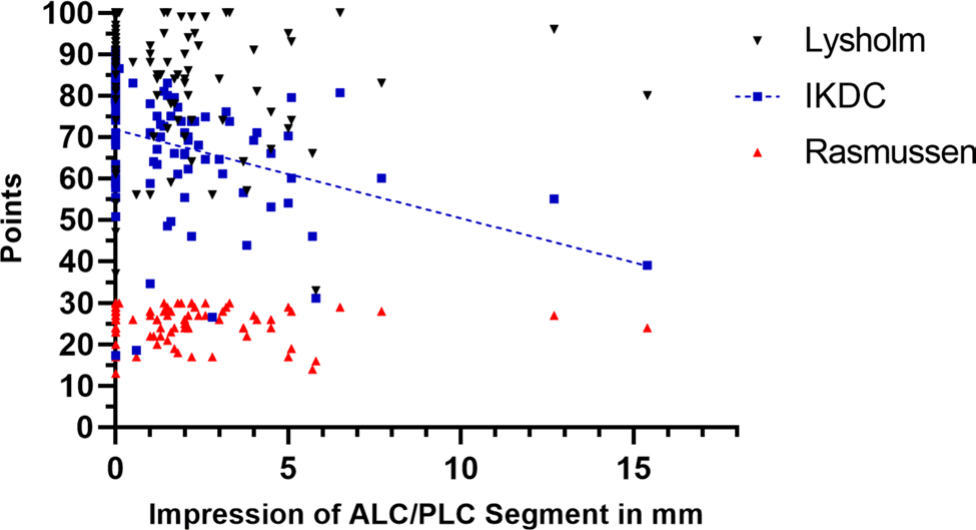
Regression PLot of Postoperative Impression between ALC/PLC Segment and clinical scores. Regression plot showing a negative association between postoperative ALC/PLC segment impression and IKDC score. The dotted line is the calculated regression line indicating a significant correlation (IKDC: *r*=-0.36 and R2 = 0.127, *p* = 0.0002). There was no postoperative correlation between ALC/PLC segment impression and Lysholm/Rasmussen score

Table 5 shows the clinical results and recorded complications. Statistically significant differences were found between the ECO and non-ECO groups regarding the IKDC score (*p* = 0.0097). There was a negative correlation between postoperative ALC/PLC segment impression and IKDC score (*r*=-0.36 and R2 = 0.127, *p* = 0.0002) (Fig. 3).

The overall incidence of postoperative complications was similar in both groups. Peroneal nerve entrapment resolved without reoperation in all cases. Two compartment syndromes were treated with fasciotomy and temporal artificial skin closure. In addition, two patients presented with arthrofibrosis and were successfully treated with arthroscopic lysis of adhesions. Revision surgery due to malreduction was deemed necessary in 5 cases, while one patient underwent an additional lateral epicondyle osteotomy where no epicondyle osteotomy had previously been performed. Five patients had persistent pain and osteoarthritis and underwent total knee arthroplasty at a mean of 18 ± 3.3 months. One patient had nonunion, which was treated with revision surgery and autologous bone grafting.

**Table 5:** Clinical outcome

Parameters	ECO (*n* = 36)	No-ECO (*n* = 46)	Total (*n* = 82)	*p*-value
Clinical Scores *				
• IKDC	71.7 ± 12.8	63.7 ± 16.1	68 ± 14.9	**0.0097**
• Lysholm	84.7 ± 12.5	83.2 ± 16.6	83.9 ± 14.6	n.s.
• Rasmussen Clinical Assessment	26.5 ± 3.0	24.9 ± 4.8	25.7 ± 4.0	n.s.
Early Postoperative Complications §				n.s.
• Revision due to malreduction > 2 mm or gap > 5 mm	3 (8.3)	2 (4.4)	5 (6.1)	
• Delayed wound healing > 7 days	5 (13.9)	2 (4.3)	7 (8.5)	
• Compartment syndrom	1 (2.7)	1 (2.2)	2 (2.4)	
• Peroneal neuropraxia	2 (5.6)	2 (4.3)	4 (4.9)	
Late Postoperative Complications §				n.s.
• Arthrofibrosis	1 (2.7)	1 (2.2)	2 (2.4)	
• TKA conversion	1 (2.7)	4 (8.7)	4 (4.9)	
• Varus instability	0	0	0	
• Nonunion	0	1 (2.2)	1 (1.2)	

*Mean ± SD, § n (%); Bold text indicating *p* < 0.05; To compare ECO and No-ECO Student`s t-Test or Kolmogorov-Smirnov`s Test was performed; TKA total knee arthroplasty

## Discussion

The study’s main finding is that patients treated with ECO achieved better clinical and radiological outcomes, even in the context of more severe injuries. These patients experienced less postoperative depression at the ALC/PLC segment junction and less angulation at the ALC/PLC and PLC/PLL segments, leading to higher IKDC scores. Higher IKDC scores were negatively correlated with the postoperative quality of ALC/PLC segment reduction, highlighting ECO’s effectiveness in more complex cases. ECO proved to be the most crucial instrument for achieving excellent fracture reduction, while different approaches did not show significant differences from one another.

Radiological and clinical data of this study undermined the concept of stepwise extension in complex lateral tibial plateau fractures, especially when evolving the posterolateral column. Submeniscal arthrotomy and fluoroscopic-assisted reduction has a significant risk of malreduction especially within the posterior quadrants [26]. While fracturoscopy and nanoscopy have been suggested, their applicability is limited in cases involving severely depressed, multi-fragmented fractures [1, 17]. Studies also found that standard approaches, such as anterolateral and modified posterolateral, do not adequately visualize the ALC/PLC intersection, which was involved in all the included fractures [1, 11]. An anterolateral approach exposes about 36% of the lateral tibial plateau’s articular surface, while an extended approach with lateral epicondylar osteotomy can expose over 83%, and with additional central subluxation of the lateral meniscus, nearly 100% can be visualized [15, 19]. Our results demonstrate that enhanced visualization of the tibial plateau, particularly the ALC/PLC and PLL, achieved with ECO, leads to improved reduction and better clinical outcomes, providing crucial clinical support for cadaveric studies that emphasize the importance of intraoperative visualization in the success of tibial plateau fracture treatments [15, 19].

The option to preoperatively plan ECO based on CT imaging, or to decide on its use intraoperatively when visualization is insufficient, offers a pragmatic approach to managing these fractures. The ECO follows the concept of the “direct approach and stepwise extension as needed” concept and can be combined with other approaches such as extended anterolateral, posterolateral or a combination of both [14].

Concerns about the potential risks associated with lateral ECO, such as inadequate consolidation leading to posterolateral rotational instability, were not supported by our findings. Furthermore, we found no significant differences in complication rates between the ECO and non-ECO groups, suggesting that ECO is a safe procedure with no increased risk of postoperative instability. However, epicondylar osteotomy should be reserved for selected complex cases. Most tibial head fractures, especially AO Type A, B1, or B2, can be managed effectively with standard approaches, making such extensive exposure unnecessary. Therefore, this technique should be used mainly when necessary to ensure precise fracture reduction under direct visual control, primarily involving the ALC, PLC, and PLL segments.

Our clinical results are in line with previous studies on the treatment of complex lateral tibial plateau fractures [6, 24, 33], though literature on the clinical outcomes of epicondylar osteotomy (ECO) is limited. Durigan et al. [8] studied a small cohort of 13 patients who underwent ECO, reporting a mean Lysholm Score of 92 ± 11.7 and a mean IKDC Score of 85 ± 12.6. The higher outcome scores in Durigan’s study may be due to the greater severity of cases in our study, which included more bicondylar split depression fractures and use of an external fixator. Korthaus et al. [16] found comparable outcomes in a case series of 10 patients, with an average Rasmussen Score of 25.0.

The MCID (minimal clinically important difference) for the IKDC Score in other studies is around 10 points, slightly exceeding the 8-point difference in our study [9, 28]. However, it varies widely (1.8–25.9 points) depending on the measurement method, and no MCID has been established for tibial plateau fractures [9]. Thus, IKDC results should be interpreted with caution. The lack of significant differences in the Lysholm score may be due to its focus on knee stability, whereas the IKDC score is more sensitive to changes in overall knee functionality [5].

Regarding surgical approaches, no significant difference was observed in subgroups between an extended anterolateral standard approach, a modified posterolateral approach, or a combination of both (see supplementary Table). This indicates that, when appropriately selected for the specific fracture type, each approach can effectively address high-grade lateral tibial plateau fractures, leading to equally favourable outcomes. This finding is particularly relevant for polytrauma patients, especially those with chest or cervical injuries, where the prone positioning required for a posterolateral approach may be disadvantageous [22].

Interestingly, the differences between ECO and no-ECO are particularly evident in the subgroups “lateral split vs. no lateral split” and “AO/OTA type B vs. C3 fractures” (see Supplementary Table 1). This may be explained by the fact that fractures with a lateral split offer a better visualization than fractures without a lateral split. Therefore, ECO may help to overcome this disadvantage, whereas in split fractures the difference is negligible. The same is true for type C fractures, where reduction is more difficult to achieve than for type B fractures. The “open book” technique, which involves an osteotomy of the lateral condyle between Gerdy’s tubercle and the tibial tuberosity while leaving the posterior hinge intact, is easily performed in the presence of a split fracture [36]. A study comparing the windowing and open-book techniques found no significant differences, which is consistent with our results in patients without ECO for lateral split fractures [36]. However, because the subgroups were relatively small (see Supplementary Table), all of these results should be treated with caution.

Another key finding is the correlation between the IKDC Score and the quality of ALC/PLC fragment reduction. This underscores the importance of precise reduction, as demonstrated by Beisemann et al. [3], who found that reductions of less than 2 mm significantly enhance clinical outcomes. Additionally, the severity of fractures, as classified by the AO/OTA system, notably affected both clinical and radiological outcomes. Our study revealed poorer clinical scores in type C fractures and significant radiological differences between type B and type C fractures. These results suggest that accurate repositioning of fracture fragments is as critical to clinical outcomes as the severity of the fracture itself, explaining why more severe fractures, which are inherently more difficult to reduce, tend to result in poorer outcomes [10, 30]. This contrasts with other studies that have suggested that injury severity does not correlate with clinical outcomes [4, 27]. This discrepancy may be explained by inappropriate classification systems, the use of a variety of different clinical scores, and the small study sizes. The time of follow-up may also be important, as osteoarthritic changes are mainly considered at later stages.

### Limitations

This study has several limitations. Firstly, the sample size included a range of complex fracture patterns, leading to some degree of heterogeneity despite rigorous inclusion and exclusion criteria that precluded matched-pair analysis. A limitation of our study is the lack of multivariable regression analysis for confounders, as the primary focus was comparing the ECO and NoECO groups, which showed no significant differences in baseline characteristics. Additionally, there was a notable decline in clinical follow-up, largely because many patients were referred from distant locations and contact information was often outdated due to relocations. The increased use of external fixation and involvement of the PLC/PLL and AC/PC segments in the ECO group likely reflects a surgical preference to use ECO in more severe fractures where improved visualization is critical, as the decision to perform ECO or not was not randomized. Furthermore, despite clinically no lateral instability was observed, varus stress radiographs were not available for these patients. Long-term outcomes, including the incidence of osteoarthritis and the potential need for conversion to total knee arthroplasty, require further investigation. Subgroup results should be treated with caution due to small subgroups.

## Conclusion

In complex lateral tibial plateau fractures, often involving the posterolateral segments, visual control of fracture reduction is the most powerful tool to achieve good and excellent clinical and radiological results. Epicondyle osteotomy has been shown to be an effective and safe procedure to visualize fracture reduction in complex tibial plateau fractures. For fractures with a deep impression of the antero-latero-central and postero-latero-central segments, a low threshold for performing an ECO is advisable. Posterolateral rim fractures may require a posterolateral approach, but otherwise an (extended) anterolateral approach is mainly used in complex lateral plateau fractures.

## Electronic supplementary material

Below is the link to the electronic supplementary material.

Supplementary Material 1

## Data Availability

No datasets were generated or analysed during the current study.
