# Supplementary material for: Lateral epicondyle osteotomy results in improved radiologic and functional outcomes in severe lateral tibial plateau fractures: a retrospective cohort study
Source: J Orthop Surg Res. 2025 Apr 10;20:363. doi: 10.1186/s13018-025-05775-3 (PMC11983902; doi:10.1186/s13018-025-05775-3)
Supplement: Supplementary file 1 — Supplementary Material 1 [file 13018_2025_5775_MOESM1_ESM.docx]

**Supplementary Table: Clinical Outcome and postoperative Reduction divided by Subgroups**

| **Subgroup** | **n** | **Depression ALC/PLC in mm** | **Angulation**  **ALC/PLC in °** | **Depression PLC/PLL in mm** | **Angulation**  **PLC/PLL in °** | **Gap in mm** | **IKDC-Score** | **Rasmussen-Score** | **Lysholm-Score** |
| --- | --- | --- | --- | --- | --- | --- | --- | --- | --- |
| **Approach** * |  |  |  |  |  |  |  |  |  |
| - Anterolateral | 76 | 1.3 ± 1.6 | 12.2 ± 15.9 | 0.9 ± 1.9 | 6.4 ± 16.1 | 2.5 ± 5.7 | 67.1 ± 16.1 | 25.5 ± 4.4 | 83.2 ± 15.8 |
| - ECO | 34 | **0.7 ± 0.9** | **7.2 ± 13.5** | 0.4 ± 1.0 | 3.0 ± 12.0 | 1.9 ± 2.8 | 69.2 ± 14.9 | 26.6 ± 3.2 | 84.0 ± 14.0 |
| - No-ECO | 42 | **2.1 ± 1.8** | **17.5 ± 16.2** | 1.4 ± 2.4 | 10.1 ± 19.2 | 3.5 ± 7.9 | 64.2 ± 17.5 | 24.3 ± 5.2 | 82.0 ± 18.0 |
| - Dorsolateral | 28 | 2.9 ± 3.8 | 18.1 ± 22.3 | 0.9 ±2.0 | 7.9 ± 19.9 | 3.4 ± 5.9 | 70.5 ± 9.1 | 25.6 ± 2.2 | 86.2 ± 10.3 |
| - ECO | 17 | **1.0 ± 1.3** | 8.6 ± 11.3 | 0.5 ± 1.7 | 2.6 ± 7.8 | 2.6 ± 6.7 | **75.3 ± 7.0** | 26.2 ± 2.6 | 85.1 ± 9.9 |
| - No-ECO | 11 | **4.2 ± 3.8** | 25.8 ± 23.3 | 1.5 ± 2.4 | 16.1 ± 29.1 | 2.7 ± 3.2 | **64.6 ± 8.9** | 27.4 ± 1.5 | 88.1 ± 10.5 |
| - Combined | 6 | 1.3 ± 1.7 | 9.7 ± 18.8 | 0.7 ± 1.0 | 0.3 ± 0.8 | 2.4 ± 3.3 | 71.9 ± 10.3 | 27.9 ± 3.0 | 91.7 ± 6.9 |
| - ECO | 5 | 1.5 ± 1.8 | 11.7 ± 20.3 | 0.9 ± 1.1 | 0.4 ± 0.9 | 2.9 ± 3.4 | 74.4 ± 8.5 | 27.2 ± 3.3 | 91.8 ± 7.7 |
| - No-ECO | 1 | 0 | 0 | 0 | 0 | 0 | 58 | 26 | 91 |
| **Lateral Split** * |  |  |  |  |  |  |  |  |  |
| - No Lateral Split | 92 | 1.5 ± 2.3 | 13.5 ± 18.2 | 1.2 ± 2.4 | 4.2 ± 12.3 | 2.7 ± 6.1 | 69.1 ± 15.0 | 25.8 ± 4.2 | 84.3 ± 14.6 |
| - ECO | 47 | **0.7 ± 1.2** | **7.3** **± 1.2** | 0.4 ± 1.3 | 6.6 ± 14.8 | 1.9 ± 5.0 | **73.1 ± 11.7** | 26.7 ±2.9 | 85.5 ± 11.9 |
| - No-ECO | 45 | **2.6 ± 2.9** | **21.4 ± 20.6** | 1.7 ± 2.8 | 2.4 ± 12.3 | 3.7 ± 7.6 | **64.2 ± 17.0** | 24.8 ± 5.0 | 82.9 ± 17.0 |
| - Lateral Split | 18 | 2.8 ± 3.1 | 15.0 ± 17.3 | 0.8 1.5 | 13.1 ± 16.2 | 2.8 ± 3.7 | 63.0 ± 13.8 | 25.3 ± 3.3 | 82.7 ± 14.9 |
| - ECO | 9 | 1.5 0.8 | 11.9 14.3 | 0.4 0.6 | 6.6 ± 14.8 | 3.0 3.4 | 64.1 16.2 | 25.2 3.6 | 80.7 15.1 |
| - No-ECO | 9 | 4.0 ± 4.0 | 18.1 ± 20.1 | 4.0 ± 4.1 | 18.1 ± 20.1 | 2.6 ± 4.1 | 62 ± 11.8 | 25.3 ± 3.2 | 84.7 ± 15.3 |
| **OTA/AO Type** * |  |  |  |  |  |  |  |  |  |
| - B3 | 54 | **1.0 ± 1.5** | 11.5 ± 15.5 | 1.1 ± 2.1 | 4.6 ± 12.5 | 1.5 ± 2.5 | **72.2 ± 14.2** | 26.4 ± 4.0 | **87.3 ± 12.6** |
| - ECO | 26 | **0.6 ± 0.8** | 7.6 ± 12.7 | 0.5 ± 1.1 | 6.1 ± 14.2 | 1.2 ± 2.3 | 75.4 ± 8.0 | 26.7 ± 2.4 | 86.6 ± 8.6 |
| - No-ECO | 28 | **1.5 ± 1.9** | 16.3 ± 17.6 | 1.8 ± 1.0 | 2.5 ± 11.3 | 1.9 ± 2.8 | 68.5 ± 18.6 | 26.0 ± 4.8 | 87.9 ± 15.6 |
| - C3 | 56 | **2.4 ± 3.0** | 15.7 ± 19.7 | 1.3 ± 2.6 | 14.1 ± 18.0 | 3.7 ± 7.3 | **64.4 ± 14.6** | 25.1 ± 4.1 | **80.8 ± 15.8** |
| - ECO | 30 | **1.1 ± 1.2** | **8.4 ± 14.4** | 0.4 ± 1.3 | 6.6 ± 14.8 | 2.8 ± 5.5 | **68.4 ± 15.3** | 26.2 ± 3.4 | 83.1 ± 15.0 |
| - No-ECO | 26 | **4.0 ± 3.6** | **24.5 ± 22.0** | 2.4 ± 3.3 | 18.1 ± 20.1 | 4.8 ± 9.0 | **59.6 ± 12.4** | 23.8 ± 4.5 | 78.2 ± 16.5 |

*Mean ± SD, Postoperative Reduction parameters (Depression ALC/PLC, Angulation ALC/PLC, Depression PLC/PLL, Angulation PLC/PLL, Gap) and outcome Scores (IKDC, Lysholm, Rasmussen) divided into subgroups (Approach, Lateral Split, OTA/AO Type). Subgroups were subdivided into fractures treated with ECO vs. No-ECO.

To compare two subgroups Student`s t-Test or Kolmogorov-Smirnov`s Test was performed. ANOVA was performed to compare more than two groups (approach) but showed no significant differences between the means. Bolt black numbers indicate a significant difference between subgroups (e.g. OTA/AO Type B vs Type C) and bolt red numbers a significant difference between ECO vs. No-ECO within the subgroup.

When comparing subgroups the IKDC- and Lysholm-Score were significantly higher in AO/OTA Type B3 fractures, while the postoperative depression of the ALC/PLC segment was significantly lower (AO/OTA B3 vs. AO/OTA C3: IKDC/Lysholm p=0.0198/p=0.0204; Impression of ALC/PLC segment p=0.01).

When comparing ECO vs. NO-ECO within subgroups (red bolt numbers) the following showed a significant difference:

Anterolateral: ALC/PLC Depression p=0.0074, ALC/PLC Angulation p=0.0007;

Posterolateral: ALC/PLC Depression p=0.0051, IKDC p=0.0015;

No Lateral split: ALC/PLC Depression p=0.0003, ALC/PLC Angulation p<0.0001, IKDC: 0.0077;

OTA/AO Type B3: ALC/PLC Depression p=0.0247;

OTA/AO Type BC: ALC/PLC Depression p=0.0390, ALC/PLC Angulation p=0.0009, IKDC p=0.0294;
